# Copy neutral absence of heterozygosity on chromosome 15 distal long arm: A surrogate marker for Prader–Willi/Angelman syndromes?

**DOI:** 10.1186/s13039-021-00558-x

**Published:** 2021-07-14

**Authors:** Veronica Ortega, Raymond J. Louie, Melanie A. Jones, Alka Chaubey, Barbara R. DuPont, Allison Britt, Joseph Ray, Scott D. McLean, Rebecca O. Littlejohn, Gopalrao Velagaleti

**Affiliations:** 1 Department of Pathology, University of Texas Health, San Antonio, TX USA; 2 Greenwood Genetic Center, Greenwood, SC USA; 3 Department of Pediatrics, University of Texas Medical Branch, Galveston, TX USA; 4 Department of Pediatrics, Baylor College of Medicine, San Antonio, TX USA; 5 Department of Molecular and Human Genetics, Baylor College of Medicine, Houston, TX USA; 6 Department of Pathology and Laboratory Medicine, University of Texas Health Science Center Mail Code, Mail Code 7750, 7703 Floyd Curl Drive, San Antonio, TX 78229 USA

**Keywords:** Copy-neutral absence of heterozygosity (CN-AOH), Uniparental disomy (UPD), Prader–Willi/Angelman syndromes, Chromosome 15 distal long arm

## Abstract

**Background:**

Copy-neutral absence of heterozygosity (CN-AOH) observed on a single chromosome or part of a chromosome may be indicative of uniparental disomy (UPD) and may require additional testing when such chromosomes or chromosome regions are known to harbor imprinted genes.

**Case presentation:**

Here we report 2 cases of neonates that presented to clinic with hypotonia, poor oral skills including inability to feed by mouth, weak cry, no response to noxious stimulation and vertical plantar creases (case 1) and hypotonia and respiratory distress (case 2). A preliminary chromosome analysis showed normal karyotypes in both cases while the high-resolution single nucleotide polymorphism (SNP) microarray showed copy neutral absence of heterozygosity involving chromosome 15 distal long arm. In case 1, the CN-AOH involved a 28.7 Mb block from genomic coordinates 73703619_102429049. In case 2, the CN-AOH involved a 15.3 Mb block from genomic coordinates 54729197_70057534. In both cases, methylation-specific PCR did not detect an unmethylated allele for the *SNRPN* gene suggesting either a deletion of paternal allele or maternal UPD for chromosome 15. Since microarray analysis did not show any copy number alterations on chromosome 15, a microdeletion was ruled out.

**Conclusions:**

Based on our cases, we suggest that CN-AOH on chromosome 15, even if it does not involve the critical region of 15q12q13, should warrant additional studies for diagnosis of Prader–Willi/Angelman syndromes.

## Background

Advent of chromosome microarray (CMA) technology, especially using the oligonucleotide probes, has revolutionized the diagnosis of copy number variation (CNV) in various conditions such as developmental delay, congenital anomalies and dysmorphic features [1]. One of these CMA platforms with the single nucleotide polymorphism (SNP)-based microarray technology that facilitates detection of CNV, but also genotype information at numerous polymorphic loci throughout the human genome. Analysis of SNP allele patterns from these SNP-based arrays cannot only confirm the CNV calls, but also can detect regions of homozygosity [2]. Due to this unique ability, SNP-based microarray analysis is becoming a more widely used diagnostic approach in many clinical laboratories [3, 4]. Regions of homozygosity observed using these SNP-based arrays are described using multiple terms such as loss of heterozygosity (LOH), absence of heterozygosity (AOH), runs of homozygosity (ROH) or long-contiguous stretch of homozygosity (LCSH) [2]. While detection of this excessive homozygosity is not diagnostic of any underlying condition and could be clinically benign, such excessive homozygosity observed on a single chromosome or part of a chromosome may be indicative of uniparental disomy (UPD) or segmental UPD. Such instances of UPD, especially in the absence of associated copy number loss is referred to as copy neutral loss of heterozygosity (CN-LOH) or in constitutional conditions as copy neutral absence of heterozygosity (CN-AOH) [5]. This CN-AOH may require additional testing when such chromosomes or chromosome regions are known to harbor imprinted genes. Here, we report on two infants who on high-resolution SNP microarray testing were found to have CN-AOH on distal long arm of chromosome 15 and further methylation-specific PCR analysis confirmed maternal UPD and clinical diagnosis of Prader–Willi syndrome.

## Materials and methods

### Case 1

A 1-week-old, Hispanic girl presented with hypotonia, poor oral skills including inability to feed by mouth, weak cry, no response to noxious stimulation and vertical plantar creases. She was born to a 43-year-old, G4P3 mother at 37.4 weeks gestation. The family history is unremarkable. The pregnancy was complicated by late entry to prenatal care at 28 weeks and oligohydramnios, but no other abnormalities were noted on ultrasound or fetal echocardiogram. At birth, the Apgars scores were 8 and 9 at 1 and 5 min, respectively. Birthweight was 2.16 kg (3rd –10th percentage for 37.4 weeks gestation) and the baby was transferred to the NICU due to low birth weight. Tests for inborn errors of metabolism, chromosome analysis, and SNP microarray were carried out in accordance with the recommended guidelines for evaluating global developmental delay [6]. An echocardiogram revealed a small secundum atrial septal defect a small anterior mid muscular type defect of interventricular septum. The infant was managed by the NICU team and a G tube placement and Nissen fundoplication was performed at 2 months of life. She was discharged at 10 weeks of life. She received occupational therapy for developmental monitoring and support.

At 2-month follow-up her weight (3.6 kg), length (51.5 cm) and head circumference (36 cm) were all below the 5th percentile. On physical examination, she had microcephaly, posteriorly rotated ears and almond shaped eyes with right eye esotropia. She continued to have poor tone and feeding. Neurologic examination showed poor proximal/shoulder girdle tone and elicited LE clonus in left leg. At 8-month follow-up, she continued to be delayed developmentally with rolling at 7 months and sitting unassisted at 8 month with no crawling. Her weight was 7.4 kg (15%ile), height was 65.2 cm (7%ile) and head circumference was 42 cm (8%ile). At this point, she was in therapy with an ECI therapist to work on gross motor skills continuing on G-tube for feeding, but her oral skills improved with therapy. Her growth showed improvement with addition of growth hormone and her feeding skills improved as well. She experienced new onset of two episodes of shaking with abnormal eye position/movement and to rule out any seizures, the infant was referred to pediatric neurology for follow-up.

## Case 2

This newborn boy was admitted to the neonatal intensive care unit for evaluation and management of hypotonia and respiratory distress. He was born to a 38-year-old gravida 5 para 3 AB 2 mother whose pregnancy was uncomplicated. The pregnancy was recognized at 8 weeks, and all prenatal ultrasounds were normal. Cesarean section delivery at 39 weeks gestation was uncomplicated. Apgar scores were 1, 5, and 8 at 1, 5, and 10 min, respectively. Birth weight was 3450 g, birth length 53 cm, and head circumference was 36 cm. Respiratory distress and severe hypotonia were evident immediately. An echocardiogram, head ultrasound, renal ultrasound, and head MRI were normal. The ammonia was 15, creatinine kinase 88, and plasma amino acids normal. Testes were not palpable. Chromosome microarray analysis and Prader–Willi syndrome methylation studies, discussed below, were arranged. Texas newborn screening, including critical congenital heart disease and hearing, was normal.

During close follow up over the next 2½ years, clinical observations confirmed the phenotype as typical for Prader–Willi syndrome (PWS). Feeding required close attention, but a gastrostomy feeding tube was not required. Features were included severe hypotonia during infancy, genital hypoplasia, cryptorchidism, small hands and feet, almond-shaped palpebral fissures, as well as gestalt recognition of Prader–Willi facies. Global developmental delays were evident. He sat unassisted at 7 months, crawled at 9 months, and walked at 18 months. Polysomnography demonstrated mild obstructive sleep apnea by 6 months. Treatment with growth hormone was begun. An orchiopexy was performed at 2 year 6 months.

The family history is positive for a maternal half-brother with autism spectrum disorder.

## Chromosome analysis

Cytogenetic analysis was carried out on peripheral blood. Culture initiation, maintenance and harvest were performed using standard methods. Chromosomes were G-banded and then analyzed using a Cytovision image analysis system (Applied Imaging, Santa Clara, CA).

## Single nucleotide polymorphism (SNP) oligonucleotide microarray

Given the complex nature of the abnormalities observed, chromosome microarray studies were carried out using Affymetrix CytoScan HD microarray. The Affymetrix CytoScan® HD Assay utilizes a high-density combined CGH and SNP array platform, which assesses approximately 2,696,550 markers, including approximately 750,000 SNP markers. Each oligonucleotide is approximately 25 base pairs long. Intragenic probe spacing is approximately 1 probe every 880 base pairs and intergenic probe spacing is approximately 1 probe every 1700 base pairs. To perform the assay, gDNA is digested with the Nsp1 restriction enzyme and digested DNA is then ligated to Nsp1 adapters. The ligation product is then amplified via polymerase chain reaction (PCR) to produce amplicons in the 200–1100 bp range. The amplicons are then purified and digested with DNAse I to produce 25–125 bp fragments. The fragments are end-labeled with a modified biotinylated base and the sample is hybridized to the array. The array is washed and stained with a streptavidin-coupled dye and a biotinylated anti-streptavidin antibody. The array is scanned with the GeneChip Scanner and the signal intensity for each marker is assessed. Using the Chromosome Analysis Suite (ChAS 3.0) software, the signal for the sample is then compared to a reference set, which is based on the average of over 400 samples. Differences in signal between the sample and reference are expressed as a log2 ratio and represents relative intensity for each marker. A discrete copy number value is determined from the relative intensity data and is displayed. Genotype information for the SNP markers is visualized with the Allele Track [7].

## Methylation specific polymerase chain reaction

After extracting the DNA from the peripheral blood, genomic DNA was subjected to bisulfite treatment and pyrosequencing using primers targeting the CpG island of the *SNRPN* gene [8, 9].

## Results

**Fig. 1:**
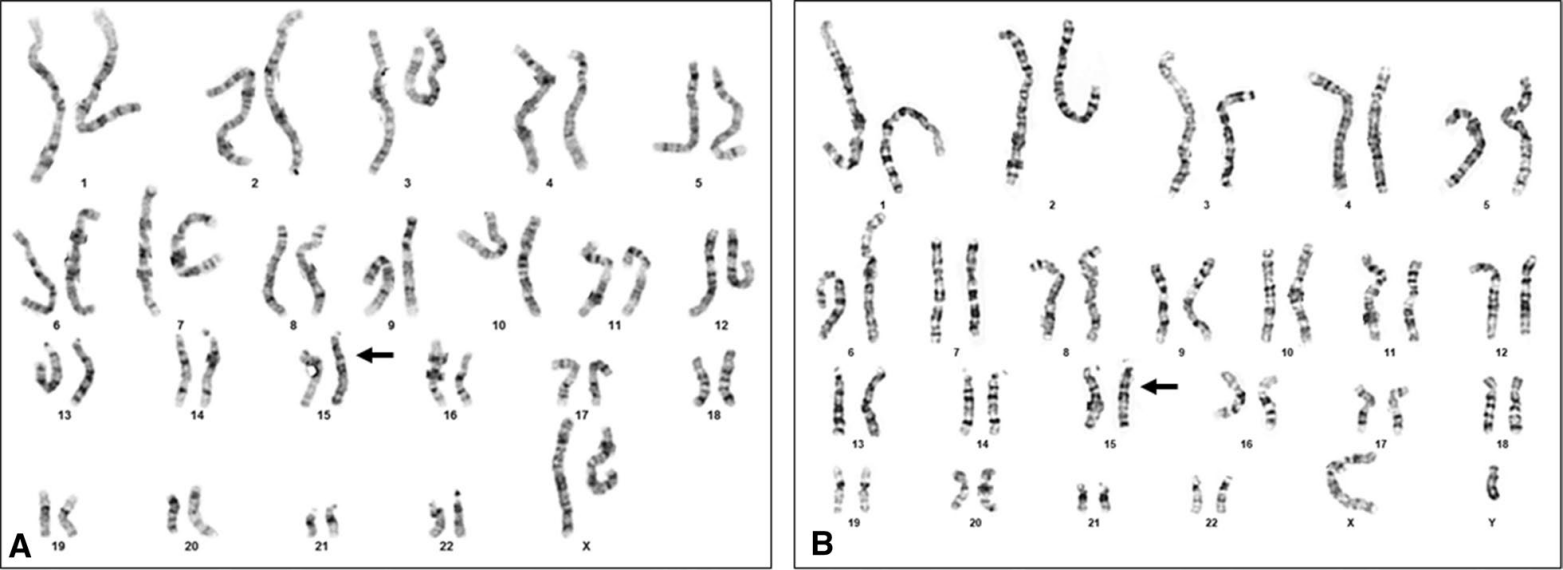
Chromosome analysis from peripheral blood showing a normal karyotype in case 1 (**a**) and case 2 (**b**). Arrow points to normal chromosomes 15

**Fig. 2:**
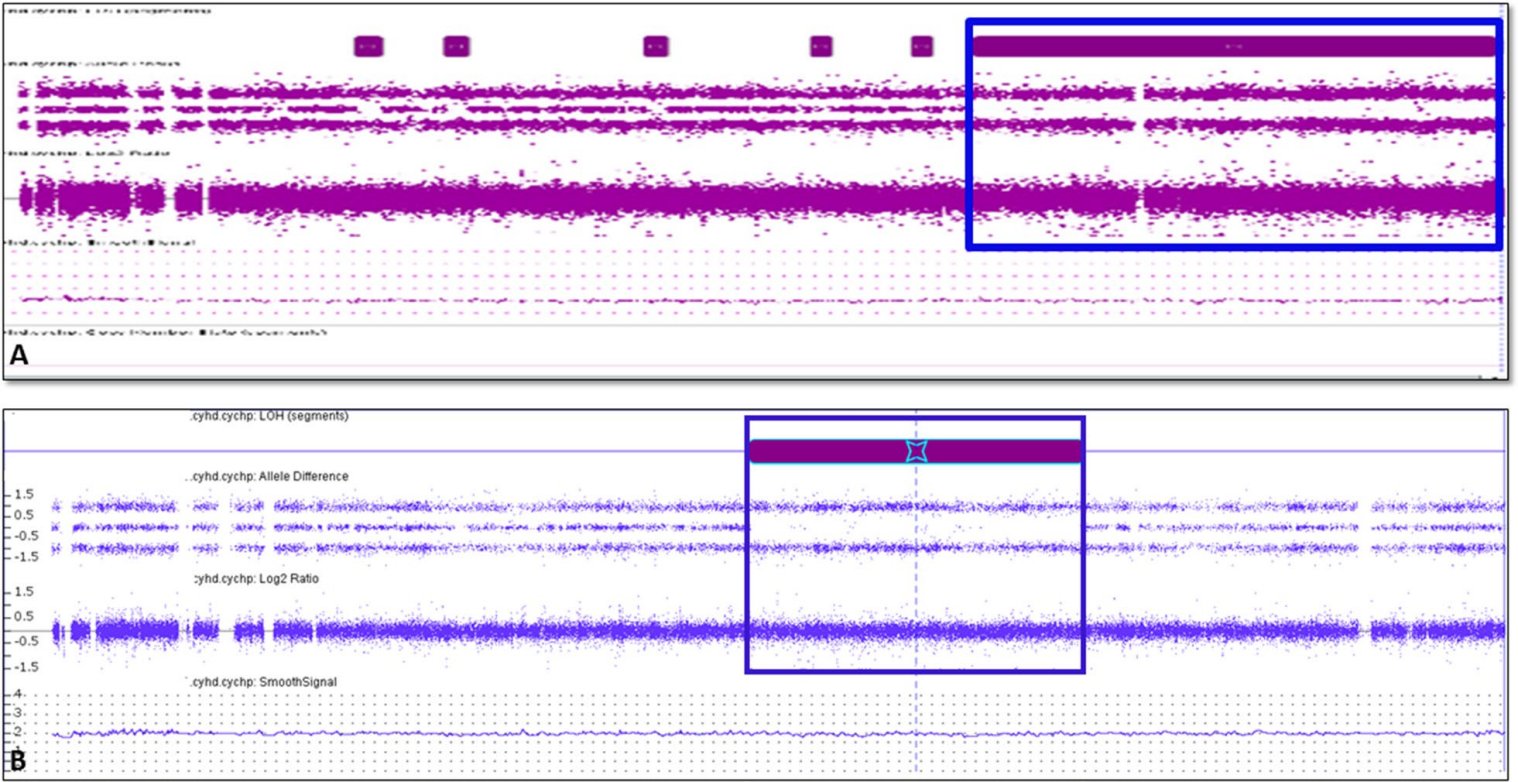
SNP-based microarray analysis showing 28.7 Mb block of AOH on distal 15q (blue box) in case 1 (**a**) and a 15.3 Mb block of AOH on distal 15q (blue box) in case 2 (**b**)

**Fig. 3:**
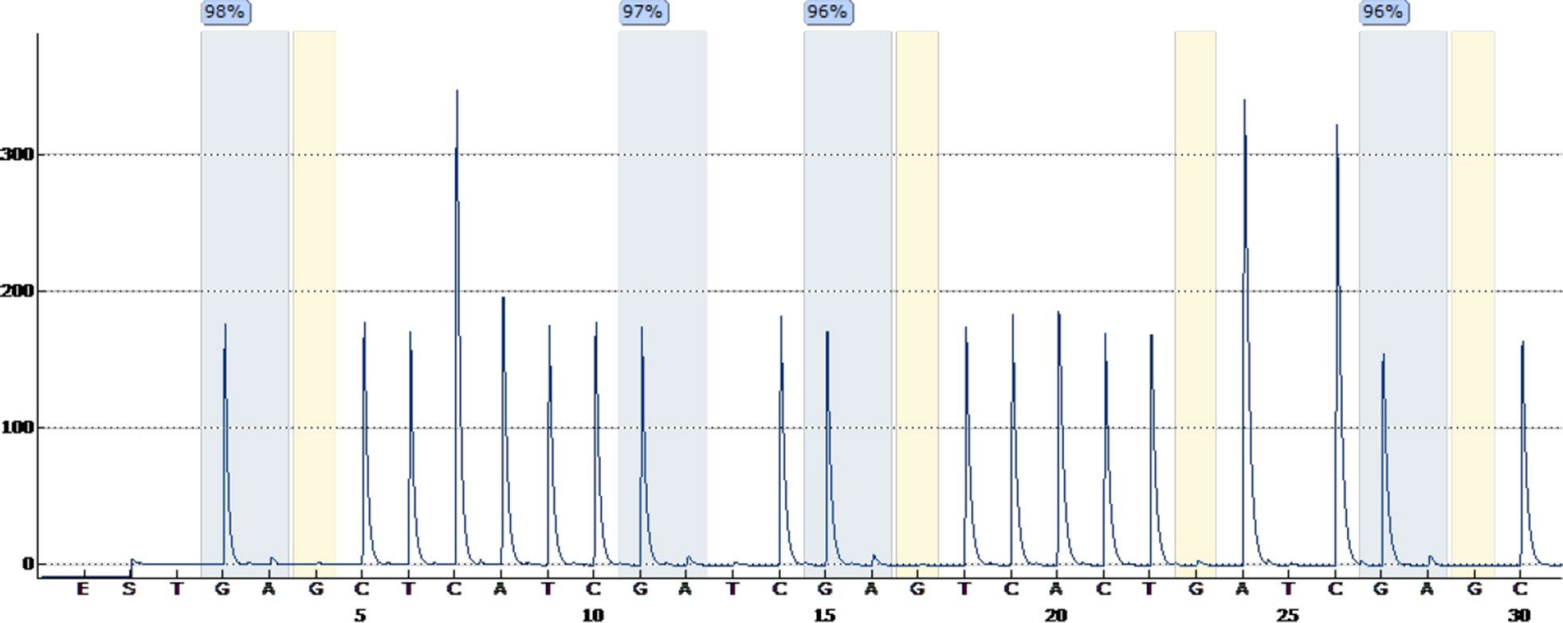
DNA methylation studies showing only methylated alleles indicating maternal UPD in case 1

Chromosome analysis showed normal karyotypes in both the cases (Fig. 1a, b). High-resolution SNP microarray results on case 1 showed an approximately 28.7 Mb block of CN-AOH on chromosome 15 distal long arm from genomic coordinates 73,703,619–102,429,049 [GRCh37] (Fig. 2a). On case 2, the SNP microarray showed a 15.3 Mb block from genomic coordinates 54,729,197_70,057,534 [GRCh37] (Fig. 2b). In both cases, this finding alone did not constitute an abnormal result and additional follow-up testing was recommended to rule out UPD. Given that the region on chromosome 15 is known to contain imprinted genes and UPD for this region can cause the Prader–Willi/Angelman syndromes, methylation studies were suggested to further characterize the region of CN-AOH observed. Methylation specific PCR studies in both cases showed the presence of methylated alleles only (Fig. 3). The presence of methylated alleles only can be indicative of either a deletion involving the paternally derived chromosome 15, or a maternal UPD or an imprinting center defect. Given that the SNP microarray showed LOH/AOH for chromosome 15 with normal gene dosage in both cases, these findings are consistent with maternal UPD 15 resulting in Prader–Willi syndrome in our patients.

## Discussion

Although Prader–Willi syndrome is a well-recognized distinct clinical entity with characteristic phenotype, a definitive diagnosis on clinical presentation alone is difficult. Clinical diagnosis of Prader–Willi syndrome in neonates is even more difficult due to the uncertainty of clinical features [10]. Hypotonia, which is the most common presenting feature in neonates with Prader–Willi syndrome, is also common in Down syndrome, thus posing a diagnostic challenge for neonatologists and pediatricians. The presence of additional congenital anomalies may help in determining the appropriate diagnosis and confirmatory laboratory testing. While karyotyping will help establish diagnosis of trisomy 21, the same is not very useful for establishing the diagnosis of Prader–Willi syndrome since karyotyping will not detect the microdeletion that accounts to about 75% of cases nor will it detect UPD that accounts for the remaining cases [11]. Because of the limitations and low resolution of karyotyping, the International Standard Cytogenomic Array (ISCA) Consortium came out with a consensus statement that chromosomal microarray (CMA) should be the first-tier clinical diagnostic test for individuals with developmental disabilities or congenital anomalies [12]. High-resolution SNP microarrays have become excellent tools in not only detecting copy number variants but also UPD by demonstrating long contiguous stretches of homozygosity limited to the chromosome 15 [4]. When such large blocks of homozygosity restricted to a single chromosome it may indicate complete isodisomy or isodisomy interspersed with heterodisomy. Since one of the limitations of SNP microarrays is the inability to detect heterodisomy, CMA can miss some cases of UPD in patients with Prader–Willi syndrome [11].

While UPD or copy number losses involving the chromosome 15q11q13 region or complete isodisomy for chromosome 15 is easy to correlate with clinical diagnosis of Prader–Willi/Angelman syndromes, UPD or CN-AOH involving distal long arm of chromosome 15 may pose a challenge for interpreting the results especially in a neonate with no obvious phenotype or minimal phenotypic features suggestive of Prader–Willi syndrome. Interestingly, previous studies reporting the diagnostic utility of SNP microarrays to detect UPD, most cases of isodisomy confirmed by SNP microarray involved distal long arm of chromosome 15 rather than proximal region [4, 13–15]. Since it is known that meiotic recombination in centromeric regions is more suppressed than telomeric regions [16], it is not surprising that these cases of segmental iso/heterodisomy for chromosome 15 shows centromeric heterodisomy while distal long arm isodisomy. Interestingly, both our patients were born to mothers with advanced maternal age (> 35 years), a known risk factor for UPD 15 [17, 18]. Further, it has been reported that advanced maternal age at child birth constitutes a risk factor for trisomy rescue/gamete complementation type UPD 15mat through meiosis 1 non-disjunction [19] which in turn often results in centromeric heterodisomy and isodisomy for the middle or distal long arm.

Although methylation studies are considered as the 1st tier test for diagnosis of Prader–Willi/Angelman syndromes [20, 21], the genetic evaluation of a neonate with generalized hypotonia depends on several factors including presence of additional congenital anomalies and/or dysmorphic features, local availability of testing, the level of diagnostic expertise of the ordering physician and cost [20, 21]. This becomes especially difficult in a pre-term neonate with no obvious clinical features suggesting a possible diagnosis of Prader–Willi/Angelman syndrome other than generalized hypotonia. Considering the specificity of the methylation studies for diagnosis of only Prader–Willi/Angelman syndromes, SNP-microarray testing may be considered in such infants to obtain broader diagnostic possibilities. While SNP-microarray studies can detect isodisomy, a significant limitation of SNP-microarray testing is the inability to detect heterodisomy resulting in missing up to 1/3rd of UPD cases [22]. However, when there are clinical features suggestive of Prader–Willi/Angelman syndromes in addition to generalized hypotonia, methylation studies must be considered the 1st tier diagnostic test.

In summary, we propose that CN-AOH observed on the distal long arm of chromosome 15 using SNP microarrays should warrant additional methylation specific-PCR studies to determine the clinical significance of such observations and correlation with clinical phenotype. CN-AOH observed on the middle or distal long arm of chromosome 15 with neonates of mothers with advanced maternal age should point to the high probability of Prader–Willi syndrome in such infants. Since early intervention is reported to benefit these children with Prader–Willi syndrome, the laboratory finding of 15q distal CN-AOH should be considered a surrogate marker for Prader–Willi syndrome and should prompt immediate follow-up confirmatory studies.

## Data Availability

All relevant data and material is included in this publication.

## Abbreviations

AOH: Absence of heterozygosity
CMA: Chromosomal microarray
CN-AOH: Copy-neutral absence of heterozygosity
ISCA: International standard cytogenomic array
LCSH: Long-contiguous stretch of homozygosity
LOH: Loss of heterozygosity
MRI: Magnetic resonance imaging
PCR: Polymerase chain reaction
PWS: Prader–Willi syndrome
ROH: Regions of heterozygosity
UPD: Uniparental disomy
